# Supplementary material for: Interventions to improve awareness and reduce the stigma associated with neurodegenerative conditions in minority ethnic communities: A scoping review protocol
Source: PLoS One. 2025 May 7;20(5):e0322009. doi: 10.1371/journal.pone.0322009 (PMC12057943; doi:10.1371/journal.pone.0322009)
Supplement: S1 File — (DOCX) [file pone.0322009.s001.docx]

**S1 File.** **Search Strategy**

| **Population one:**  “minority ethnic*” OR “ethnic minorit*” OR BAME OR BME OR CALD OR “cultural diver*” OR culture* OR “South Asia*” OR Indian* OR Pakistani* OR Bangladeshi* OR Gujarati* OR Punjabi* OR Sikh* OR Muslim* OR Hindi* OR Black* OR Africa* OR Caribbea* OR Racial OR Race OR migra* |
| --- |
| **Population two:**  dement* or Alzheimer* or Parkinson* or Lewy or Fronto* or Huntington or Chorea or “amyotrophic lateral sclerosis” or ALS or “motor neuron* disease” or MND or “progressive muscular atrophy” or Gehrig or neurodegen* or neurolog* or “cognitiv* impair*” |
| **Intervention:**  education* OR aware* OR “health promot*” OR “health educat*” OR “mass media” OR “campaign*” OR book* OR leaflet* OR information OR DVD OR “social media” OR “community workshop*” |
| **Outcomes:**  Attitude* OR awareness OR confidence OR “help seeking” OR knowledge OR stigma OR “self-efficacy” |
