# Supplementary material for: Interventions to improve awareness and reduce the stigma associated with neurodegenerative conditions in minority ethnic communities: A scoping review protocol
Source: PLoS One. 2025 May 7;20(5):e0322009. doi: 10.1371/journal.pone.0322009 (PMC12057943; doi:10.1371/journal.pone.0322009)
Supplement: S3 File — (PDF) [file pone.0322009.s003.pdf]

NIHR Policy Research Unit in Dementia and Neurodegeneration, University of Exeter (known as DeNPRU Exeter)

Equality Impact Assessment Form

An equality impact assessment (EIA) is an evidence-based approach designed to help organisations ensure that their policies, practices, events and decision-making processes are fair, do not present barriers to participation and do not disadvantage any protected groups from participation (UKRI, 2022)

1. Contact Details

Please complete your personal details and contact information in the spaces provided.	
Impact Assessor's Full Name	Project Lead: Dr Sahdia Parveen (University of Bradford).<br>Project Team: Prof Jan Oyebode, Prof Karen Windle and Dr Maria Caulfield (University of Bradford).<br>Prof Linda Clare and Dr Catherine Charlwood (University of Exeter).<br>Prof Matthew Prina (Newcastle University)<br>Dr Adelina Comas-Herrera (LSE)
University	University of Bradford
Email	s.parveen27@bradford.ac.uk
Completion Date	15 April 2024

2. About the policy/ research activity

**Please describe what you are impact assessing**	
Title	Reducing inequalities across the care pathway for minority ethnic communities affected by dementia or other neurodegenerative conditions
Description / Purpose (Summary of main aims and objectives)	The main aims of this project are as follows:<br>1. How we can raise awareness of and reduce the negative attitudes associated with neurodegenerative conditions (for example, Dementia, Parkinson's Disease, Huntington's Disease and Motor Neurone Disease) in South Asian and African Caribbean communities in England.

	2. How we can improve how neurodegenerative conditions are diagnosed and how the diagnosis is explained to South Asian and African Caribbean communities.<br><br>3. How we can improve access to treatment and support for South Asian and African Caribbean communities.<br><br>This project will involve a number of activities to address the above aims and objectives:<br>1. A scoping review of the literature followed by roundtable events with stakeholders (aim 1).<br>2. Focus groups with health care professionals (aim 2)<br>3. A review of reviews and roundtable events with stakeholders (aim 3).<br><br>The wider policy research unit will be supported by active PPIE (Patient and Public Involvement and Engagement) via a core group of 16 individuals with each project (with a total of 8-12 projects across the PRU) being supported by two PPIE members. Please note separate equality impact assessments (EIA) are being carried out for each project. This EIA is specifically for the 'Reducing inequalities across the care pathway for minority ethnic communities affected by dementia or other neurodegenerative conditions' project being led from University of Bradford.

Which groups will be affected by this policy/research activity?
**Whilst this project will focus on South Asian and African Caribbean communities, we have assessed the impact of the project activities on all of the protected characteristics.**

3. Description of impact / potential impact

What consultation (PPIE) work has been done in relation to this policy/research activity?
This project is currently being supported by a South Asian female carer who provided support for a parent living with dementia and a South Asian person living with Parkinson's Disease who is

supporting a parent with advanced Vascular Dementia. The two PPIE members have been involved with discussions as the project questions have been developed and will be actively involved in supporting research activities such as planning roundtable stakeholder events. We are conscious that our PPIE attached to this project does not yet include representation from African or Caribbean communities and we are currently engaging with various charity organisations to identify potential contributors.

**Based on your research / evidence, which groups might this policy or research affect more or less than others?** Ethnicity/Race, Age, Disability, Gender, Religion/Belief, Maternity/Paternity, Sexual orientation, Socio economic status, Caring responsibilities	
Have you analysed equality data for each of the groups identified above?	Yes **X** Please note that there is a lack of accurate ethnicity data and recent reports from the Race Equality Foundation[1] have suggested that ethnicity data is poorly collected and reported in health care. Furthermore, there is little consensus on expected prevalence of dementia and neurodegenerative conditions in South Asian and African Caribbean communities.<br>No ☐
Have you identified / researched anecdotal or alternative evidence?	Yes **X**<br>No ☐
Have you attached the evidence to this impact assessment?	Yes **X**<br>No ☐

4. Description of Evidence

Please provide evidence of impact on equality groups in the table below.

**What does your evidence tell you?**			
**Equality Group**	**Nature of impact (Positive / Negative / Neutral / Impact not known)**	**Using examples from the evidence you have collected, please describe the positive or negative impact on the equality groups you have identified in this section**	**Action to address negative impact**
Age	**Negative**	There is evidence to suggest that receipt of support services is lower amongst older people (and women)[2]. The workshops will take place online and older people may be disadvantaged if unfamiliar with technology.	Information will be provided to attendees on how to access the Teams/Zoom meetings. Support will be provided to people unfamiliar with the platforms (practice run throughs). Participants will also be given the

			option to participate in a one to one phone call if preferred.
Disability	**Negative**	Online can be helpful for people with some disabilities to attend, although those with communication difficulties may struggle, and we need to be mindful of this. The Zoom platform is preferable to Teams.	Attendees will be asked if they have any requirement/need for adjustments to be made and this will be addressed.<br><br>People who are deaf may still be excluded due to the unavailability of funded interpreters, but can still be included if there is a significant other who is able to support the participation as an interpreter.
Gender	**Positive**	There is evidence that women ae disproportionately affected by dementia,[3] therefore may benefit from the findings from this project.	We will offer single gender space during the online workshops. We will also collect demographic information prior to the workshops.
Marriage and Civil Partnership	**Neutral**	People who are married or in a civil partnership are less likely to develop dementia (Sommerlad et al, 2018).[4]<br><br>People who are married or in a civil partnership may also be providing care therefore there is an overlap with the protected characteristic caregiving responsibilities.<br><br>Some intervention delivery and/or evaluation may rely on the involvement of family members in different capacities. Lack of extended family, or even minimal family support, can pose barriers to accessing timely support or services. There is some evidence from the IDEAL project that people living alone, particularly older women, are less likely to access services due to various factors.[5]	

Race/ Ethnicity/Religion	**Positive and negative**	People from minoritized ethnic groups may experience barriers to participate in research related activities, such as PPIE (Waheed et al., 2020).[6] We will aim to minimize these barriers by linking in with community organisations, using translators and providing flexible options of participation (in person or online, group or individual).<br><br>The scoping review has identified some statistics on prevalence rates of neurodegenerative conditions across various ethnicities and races. There is debate as to whether the low numbers of people diagnosed with dementia and other neurodegenerative conditions from minority ethnic communities is due to lower risk or people not seeking a diagnosis due to the stigma attached to the conditions.	For the literature reviews, actions to address impact will include checking the ethnicity of the participants, where reported, and not be misled by the religion reported. A number of papers have been found to report the individual's religion as opposed to ethnicity.<br><br>For focus groups or workshops, it's crucial to consider religious holidays and times of religious observance. For example, we would avoid scheduling events on Friday afternoons due to the prayer times for Muslims.<br><br>The focus group recruitment strategy aims to purposively target professionals with experience in working with minority ethnic communities and those with limited knowledge to consider differences in approaches to practice and what works well etc.<br><br>Given the considerable diversity within South Asian and African Caribbean or Black communities, we anticipate challenges in engaging a broad spectrum of individuals. We have therefore identified key professional allies

			to help us make contacts and engage with communities of interest.<br><br>We will also make use of interpreters where needed.<br><br>Build trust and rapport with communities (Parveen et al., 2018)[6]
Sexual Orientation	**Positive and negative**	Conditions such as dementia may have specific implications for those from LGBTQ+ communities for example memory problems might make it harder for you to remember who you have told about your sexual orientation or gender identity. There are fewer support services for those from LGBTQ+ affected by dementia and other neurodegenerative conditions, therefore the views of this community will be important within this project.	It is important to gather this information sensitively using demographic questionnaires before conducting any focus groups or workshops, allowing individuals the chance to disclose their sexuality privately, if they choose to do so.
Maternity/Paternity	**Neutral**	Paternity leave is often overlooked in research.	We will be open to receiving comments and feedback via email instead of workshop attendance and will be flexible with time scales.
Socio Economic status	**Negative**	People who experience socioeconomic disadvantage are at higher risk of dementia (Alzheimer's Association®, 2022).[7]<br><br>As with people from minority ethnic backgrounds, people from disadvantaged backgrounds may experience more barriers to participate in the research, for example, due to expenses associated with travel, not being able to participate due to long working hours.	On balance, online groups may offer the most practical approach in terms of time, resources, and reach. However, if needed, one-on-one, in-person discussions can be arranged for people who wish to participate but are unable to join online.<br><br>It will be important to establish ground rules during workshops to ensure that everyone has an equal

		From our experience of conducting research using virtual platforms, people from lower (Socio Economic Status) or those working as health care assistants are less likely to have access to laptops and are more likely to join via their phone. There may be issues with wi-fi.	opportunity to speak and feels comfortable participating, regardless of their seniority. We will not ask people to share their role or use titles so participants do not feel intimidated by those perceived as more senior.<br><br>We will collect demographic information prior to the events. This approach may help mitigate intimidation and create a more equal platform from which everyone can speak honestly.
Caring responsibilities	**Positive and negative**	Unpaid carers, including spouses and adult children, encounter diverse challenges attending workshops due to their employment or intensity of their caregiving responsibilities/routines.	After consultation with carers, there may be a suitable window between 1-3pm that has been identified as convenient for some unpaid carers, while others, especially those still employed, may prefer early evening sessions.<br>The unpredictable nature of caregiving means that last minute changes in plans are common. Therefore, it will be important to have alternative ways (such as telephone calls) that unpaid carers can still participate if they cannot attend on the day but would still like to contribute to the research and feel included.

5. EIA Outcome and Action Planning

**If the impact of the policy / research activity is negative for any of the groups identified above what actions will you take to address this?**		
**Action**	**Timescale**	**Responsibility**
Scoping review: Check ethnicity whilst screening	Feb 2024-June 2024	Sahdia and Maria
Create an invitation letter and outline support for accessibility and any other needs. Set up calls with people who need additional support	August 2024	Maria and Cathy with input from wider team
Create demographics form to be sent out prior to workshop attendance	September 2024	Research fellows with input from wider team
Liaise with experts by experience to set up workshop dates and times that will be appropriate for diverse range of groups	August 2024	Maria, Rachael
Engage with key professional allies to connect with diverse communities	June-Aug 2024	Sahdia, Cathy, research fellows
Arrange for interpreters if required	Aug-Oct 2024	Maria/second research fellow
Arrange for one to one calls with participants if needed	Aug-Oct 2024	Maria/second research fellow
Set up 'ground rules' to ensure sensitivity and inclusion during workshops	Aug 2024	Maria

Final Decision	Tick	Justification/Explanation
No negative impact identified, therefore activity will proceed as planned.		
Activity will be adapted to in a way you think will eliminate bias		
Do not proceed with activity as data suggests significant negative impact on protected groups		
Barriers and impact identified, however having considered all	X	

available options carefully, there appear to be no other proportionate ways to achieve the aim of the policy or research activity (e.g. in extreme cases or where positive action is taken). Therefore, you are going to proceed with caution with this policy or research activity knowing that it may favour some people less than others, providing justification for this decision.		

References

1. Improving the recording of ethnicity in health datasets. Exploring the views of community respondents and the healthcare workforce (2022). Race Equality Foundation. https://raceequalityfoundation.org.uk/wp-content/uploads/2023/01/CC165_REF_Wellcome_Trust_Report_FINAL.pdf
2. Van Horik, J., Collins, R., Martyr, A., Henderson, C., Jones, R.W., Knapp, M., Quinn, C., Thom, J., Victor, C., & Clare, L., on behalf of the IDEAL programme team (2022). Limited receipt of support services among people with mild-to-moderate dementia: findings from the IDEAL cohort. International Journal of Geriatric Psychiatry, 37. DOI: 10.1002/gps.5688
3. Women and Dementia: A global research review (2015) Alzheimer's Disease International. https://www.alzint.org/resource/women-and-dementia-a-global-research-review/
4. Sommerlad, A., Ruegger, J., Singh-Manoux, A., Lewis, G., & Livingston, G. (2018). Marriage and risk of dementia: systematic review and meta-analysis of observational studies. Journal of neurology, neurosurgery, and psychiatry, 89(3), 231–238. https://doi.org/10.1136/jnnp-2017-316274
5. Clare, L., Martyr, A., Henderson, C., Gamble, L., Matthews, F.E., Quinn, C., Nelis, S.M., Rusted, J., Thom, J., Knapp, M., Hart, N., & Victor, C. (2020). Living alone with mild-to-moderate dementia: findings from the IDEAL cohort. Journal of Alzheimer's Disease, 78, 1207-1216. DOI: 10.3233/JAD-200638
6. Waheed, W, Mirza, N, Waheed, MW, et al. Recruitment and methodological issues in conducting dementia research in British ethnic minorities: A qualitative systematic review. Int J Methods Psychiatr Res. 2020; 29:e1806. https://doi.org/10.1002/mpr.1806
7. Alzheimer's Association® (2022). Lower Socioeconomic Status in Childhood, Persistent Low Wages Linked to Risk for Dementia and Faster Memory Decline. Accessed at https://aaic.alz.org/releases_2022/lower-socioeconomic-dementia-risk.asp
