# Supplementary material for: Interventions to improve awareness and reduce the stigma associated with neurodegenerative conditions in minority ethnic communities: A scoping review protocol
Source: PLoS One. 2025 May 7;20(5):e0322009. doi: 10.1371/journal.pone.0322009 (PMC12057943; doi:10.1371/journal.pone.0322009)
Supplement: Attachment — Submitted filename: Response to Reviewers.docx [file pone.0322009.s006.docx]

# Response to reviewers

Dear Vincenzo De Luca and the reviewer,

Thank you for taking the time to review our manuscript and for providing your feedback. We have reviewed the manuscript in accordance with PLOS ONE’s style requirements, including those for naming files, and have made the necessary adjustments.

To improve the clarity of our protocol, we have carefully considered your suggestions. Our detailed responses to the three areas identified are outlined below.

| **Reviewer comments** | **Authors responses** |
| --- | --- |
| 1. The definition of "culturally tailored interventions" is lacking in the manuscript. A more detailed definition should be provided to help readers understand what the authors are talking about. Or the authors can provide some examples of such interventions. If the authors can provide some quantitative criteria to define "culturally tailored interventions", that would be even better. | On page 6, line 117, we have expanded our definition of an ‘intervention’ and provided an example to illustrate what a culturally tailored intervention might entail. |
| 2. The inclusion of grey literature is unclear. Google search can be biased. A better, more objective criterion should be set to address this issue. | On page 9, line 188, we have outlined our approach to searching the grey literature as explicitly as possible. We believe our method is comprehensive and thorough compared to many other scoping reviews, which often only state that a search of the grey literature was conducted without providing further detail. We will adopt a two-pronged approach.  First, we will use advanced Google search parameters on a preselected list of 77 websites. The list of websites and an example search string are provided. Since the most relevant PDFs are typically prioritised in Google search results, we will download and screen the first 20 PDFs returned for each website.  Second, we will conduct a broader Google web search. We have specified the key terms and pairings we will use, as well as the number of search results we will review.  Together, these methods will help identify a diverse range of grey literature sources, including PDFs, videos, podcasts, webinars, and other online resources. |
| 3. The inclusion criteria of stakeholder input might be too limited. I understand that policy makers, practitioners and the minority people should be included. But representatives from the major public should also be included, as the intervention will inevitably show up in public and it's a must to consider if the intervention can also be not just accepted by the major public and even spread/shared by them to other people. These types of communication will accelerate/improve the impact of such interventions in the whole society. | As this is the first workstream of a policy research project, our stakeholder focus is experts-by-experience who have lived experience of neurodegenerative conditions and those already working with or for ethnic minority communities affected by neurodegenerative conditions. Experts-by-experience who have already been affected by awareness campaigns, for better or worse, are in a strong position to advise on what works. Many of them have been actively involved in raising awareness within their own communities since being affected by neurodegeneration and so again have important perspectives to share. Given that experts-by-experience represent different conditions, we seek to uncover ways in which awareness raising is working for certain conditions and where best practice might be adopted for others.  Local/regional practitioners and national Equality, Diversity, and Inclusion (EDI) leads (or those with EDI as part of their portfolio) will be consulted as they have a real-time view of what is and is not working on the ground and the scale of the problem and can share insights into efforts which have yielded positive results, and wrong turns taken. UK national leads for EDI are in positions to create policy changes for their organisations and influence public perceptions.  As the focus of this project was never to identify and promote one single awareness intervention, the broader public will not be consulted in this instance. However, the authors would strongly recommend that any original research investigating the feasibility of implementing particular interventions include this group in their stakeholder consultations. |

Please note some further changes made to the manuscript.

| Page, 4, line 90 | We have clarified the definition of a minority ethnic group: *A minority ethnic group is a group of people who share a common cultural identity, traditions, and languages, distinguishing them from the majority ethnic group in a particular country.* |
| --- | --- |
| Page 8, line 174 | We have presented the inclusion and exclusion criteria in a table to make the information easier for the reader |
| Page 13, line 268 | We have presented the potential outline for the consultation workshops as a figure (Fig 1. Potential outline for consultation workshops) |
